# Prognostic Impact of Lymphocytes in Soft Tissue Sarcomas

**DOI:** 10.1371/journal.pone.0014611

**Published:** 2011-01-27

**Authors:** Sveinung W. Sorbye, Thomas Kilvaer, Andrej Valkov, Tom Donnem, Eivind Smeland, Khalid Al-Shibli, Roy M. Bremnes, Lill-Tove Busund

**Affiliations:** 1 Department of Clinical Pathology, University Hospital of North Norway, Tromso, Norway; 2 Institute of Medical Biology, University of Tromso, Tromso, Norway; 3 Department of Oncology, University Hospital of North Norway, Tromso, Norway; 4 Institute of Clinical Medicine, University of Tromso, Tromso, Norway; 5 Department of Pathology, Nordland Central Hospital, Bodo, Norway; Universidade de São Paulo, Brazil

## Abstract

**Purpose:**

The purpose of this study was to clarify the prognostic significance of lymphocyte infiltration in soft tissue sarcomas (STS). Prognostic markers in potentially curable STS should guide therapy after surgical resection. The immune status at the time of resection may be important, but the prognostic significance of tumor infiltrating lymphocytes is controversial as the immune system has conflicting roles during cancer development.

**Experimental Design:**

Tissue microarrays from 249 patients with STS were constructed from duplicate cores of viable and representative neoplastic tumor areas. Immunohistochemistry was used to evaluate the CD3+, CD4+, CD8+, CD20+ and CD45+ lymphocytes in tumors.

**Results:**

In univariate analyses, increased numbers of CD4+ (P = 0.008) and CD20+ (P = 0.006) lymphocytes in tumor correlated significantly with an improved disease-specific survival (DSS) in patients with wide resection margins (n = 108). In patients with non-wide resection margins (n = 141) increased numbers of CD3+ (P = 0.028) lymphocytes in tumor correlated significantly with shorter DSS. In multivariate analyses, a high number of CD20+ lymphocytes (HR = 5.5, CI 95%  = 1.6–18.6, P = 0.006) in the tumor was an independent positive prognostic factor for DSS in patients with wide resections margins.

**Conclusions:**

High density of CD20+ lymphocytes in STS with wide resection margins is an independent positive prognostic indicator for these patients. Further research is needed to define if CD20+ cells can modify tumors in a way that reduces disease progression and metastatic potential.

## Introduction

Soft tissue sarcomas (STS) are relatively rare, heterogeneous malignancies of mesenchymal origin with a high mortality rate. They comprise less than 1% of adult malignancies[1] and approximately 50% of the STS patients will succumb to their disease because of metastasis or local relapse[2]. There are several prognostic factors which determine tumour progression, and ultimately the patient's outcome, including positive resection margins; presence of local recurrence; and tumour grade, size, location, depth and histological entity[3]–[9].

Many studies have been designed to investigate the prognostic factors of STS by using immuno-histochemical methods[10]. Most of the published data have focused on the expression of markers for cell kinetics and regulatory proteins of the cell cycle.

Tumor infiltrating lymphocytes are considered to be an indication of the host immune reaction to tumor antigens[11], and their clinical significance has been reported in a variety of human solid tumors.

CD3 is a part of the T cell receptor (TCR) complex on a mature T lymphocyte. CD4 is a glycoprotein expressed on the surface of T helper cells, regulatory T cells, monocytes, macrophages, and dendritic cells. CD8 is a transmembrane glycoprotein that serves as a co-receptor for the T cell receptor (TCR). Like the TCR, CD8 binds to a major histocompatibility complex (MHC) molecule, but is specific for the class I MHC protein. CD20 is a non-glycosylated phosphoprotein expressed on the surface of all mature B-cells. CD20 is expressed on all stages of B cell development except the first and last; it is present from pre-pre B cells through memory cells, but not on either pro-B cells or plasma cells. The CD45 antigen was originally called leukocyte common antigen. The protein encoded by this gene is a member of the protein tyrosine phosphatase (PTP) family. This gene is specifically expressed in hematopoietic cells. This PTP has been shown to be an essential regulator of T- and B-cell antigen receptor signalling (http://www.genecards.org).

The purpose of this study was to clarify the prognostic significance of lymphocyte infiltration in non-gastrointestinal stromal tumor (GIST) STSs. To achieve this, we analyzed the expression of CD3+, CD4+, CD8+, CD20+ and CD45+ lymphocytes in 249 patients with non-GIST STS in relation to other clinicopathological variables.

## Materials and Methods

### Patients and Clinical Samples

The National Cancer Data Inspection Board and The Regional Committee for Research Ethics approved the study. The Regional Committee approved that written consent from the patients for their information to be stored in the hospital database and used for research was not needed. This because most of the material was more than 10 years old, and most of the patients being dead. The material was collected from our approved biobank for paraffin embedded material and slides. Data were analyzed anonymously.

Primary tumor tissues from patients diagnosed with STS at the University Hospital of North Norway (UNN) from 1973 to 2006 and the Hospitals of Arkhangelsk region, Russia, were used in this retrospective study. 496 potentially suitable patient records were identified from the hospital database but only 249 of these were eligible for this study because they had complete medical records and adequate paraffin-embedded tissues blocks. This report includes follow-up data for 167 Norwegian patients and 82 Russian patients up to September 2009. The median follow-up was 38 (range 0–392) months. Complete demographic and clinical data were collected retrospectively. Formalin-fixed and paraffin-embedded tumor specimens were obtained from the archives of the Departments of Pathology at UNN and Archangelsk. The tumors were graded according to the French Fèdèration Nationales des Centres de Lutte Contre le Cancer (FNCLCC), [WHO Tumors of Soft Tissue and bone, 2002]. Wide resection margins were defined as wide local resection with free microscopic margins or amputation of the affected limb or organ. Non-wide resection margins were defined as either marginal or intralesional resection margins, or no surgery.

### Microarray construction

The histology of all soft tissue sarcoma cases were reviewed by two pathologists (AV and SWS). Tissue microarrays (TMAs) were constructed for high-throughput molecular pathology research[12]. The most representative areas of viable tumor cells were carefully selected and marked on the hematoxylin and eosin (HE) slides for the corresponding donor blocks and sampled for the tissue microarray collector blocks. The TMAs were assembled using a tissue-arraying instrument (Beecher Instruments).

Studies suggest that punching multiple 0.6 mm cores from different regions captures the heterogeneity of the tumors more accurately than single 2 to 4 mm core[13]. Hence, we chose using two 0.6-mm cores of viable neoplastic tissue that were selected to be as representative as possible (different areas), after reviewing all original sections of the tumor and taking the heterogeneity in consideration. To include all core samples, 12 tissue array blocks were constructed. Multiple 4-µm sections were cut with a Micron microtome (HM355S) and stained by specific antibodies for immunohistochemistry (IHC).

### Immunohistochemistry (IHC)

The applied antibodies were subjected to in-house validation by the manufacturer for IHC analysis on paraffin-embedded material. Ventana Benchmark, XT automated slide stainer (Ventana Medical System, France) was used for IHC. Sections were deparaffinized with xylene and rehydrated with ethanol. Antigen retrieval was performed by placing the specimens in 0.01 M citrate buffer at pH 6.0 and exposing them to two repeated microwave heatings of 10 minutes each at 450W. The DAKO Envision+ System-HRP (DAB) kit was used as endogen peroxidase blocking. As negative staining controls, the primary antibodies were replaced with the primary antibody diluents. Primary mouse monoclonal antibodies were incubated for 16 minutes (CD20, clone L26 Ventana), 20 minutes (CD4, clone 1F6 Novocastra, dilution 1∶5) and 32 minutes (CD8, clone 1A5 Ventana) at room temperature. The Ventana antibodies were pre-diluted from the manufacturer. Biotinylated goat anti-mouse IgG and mouse anti-rabbit IgM were used as secondary antibodies. The DAB was used to visualize the antigens. This was followed by application of liquid diaminobenzidine and substrate-chromogen, yielding a brown reaction product at the site of the target antigen. Finally, slides were counterstained with hematoxylin to visualize the nuclei. For each antibody, including negative controls, all TMA staining was performed in a single experiment.

### Scoring of IHC

**Figure 1:**
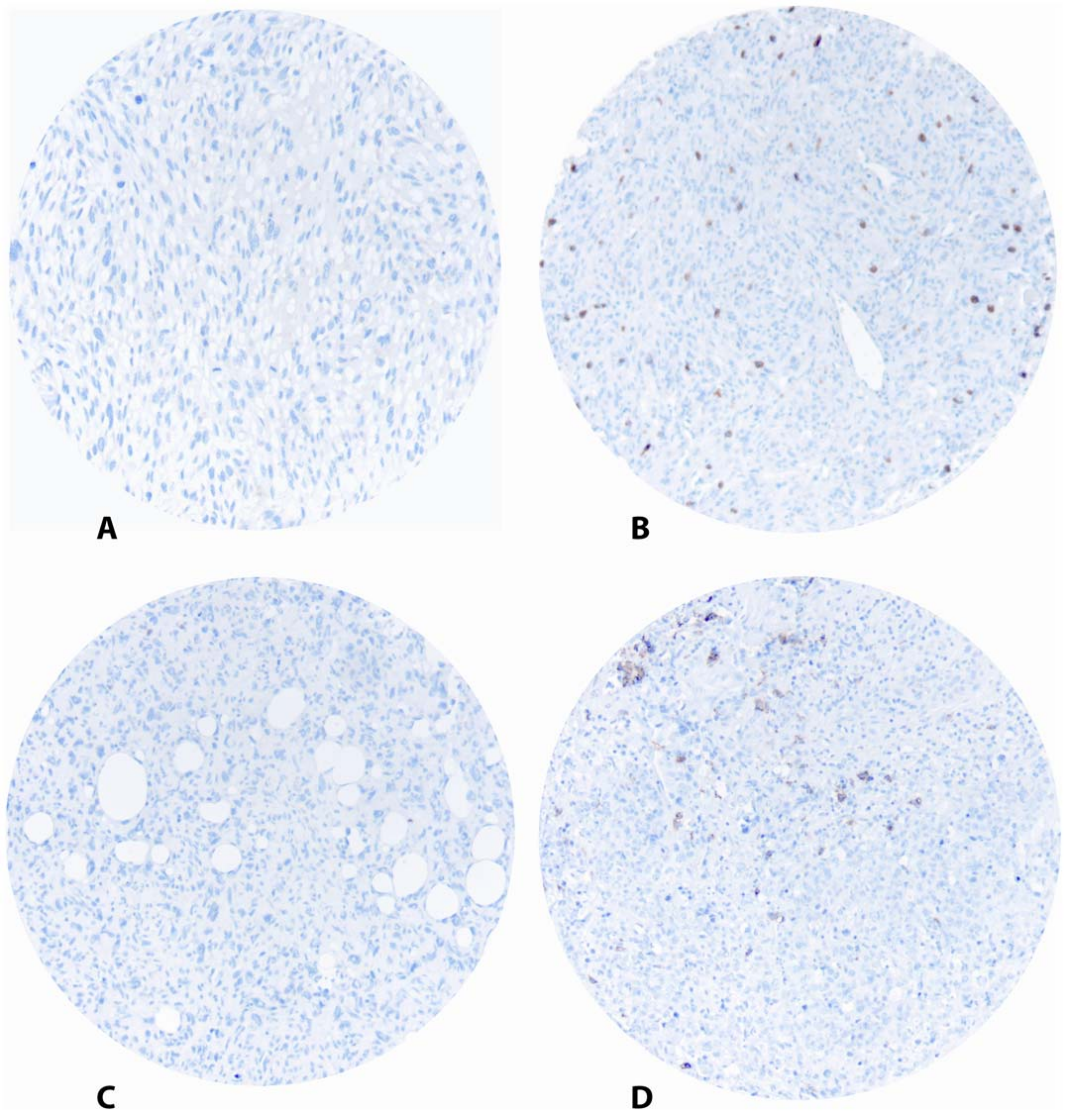
IHC microscopic pictures of TMA of soft tissue sarcoma representing different scores for CD4+ and CD20+ lymphocytes. (A) CD4 low score; (B) CD4 high score; (C) CD20 low score; (D) CD20 high score. Original magnification X 400.

The ARIOL imaging system (Genetix, San Jose, CA) was used to scan the slides for antibody staining of the TMAs. The specimens were scanned at a low resolution (1.25×) and high resolution (20×) using an Olympus BX 61 microscope with an automated platform (Prior). The slides were loaded in the automated slide loader (Applied Imaging SL 50). Representative and viable tissue sections were scored manually on a computer screen semi-quantitatively for cytoplasmic staining. Tumors were scored as 0 (no cells), 1 (1–5 cells), 2 (6–19 cells) or 3 (20+ cells) (Figure 1). All samples were made anonymous and independently scored by two pathologists (AV and SWS). Where there was disagreement, the slides were re-examined and a consensus was reached by the observers. When assessing a variable for a given score, the scores of the other variables and the outcome were hidden from the observers.

### Statistical Methods

All statistical analyses were done using the statistical package SPSS (Chicago, IL), version 16. The immunohistochemistry scores from each observer were compared for interobserver reliability by use of a two-way random effect model with absolute agreement definition. The intraclass correlation coefficient (reliability coefficient) was obtained from these results.

The Chi-square test and Fishers Exact test were used to examine the association between molecular marker expression and various clinicopathological parameters. Univariate analyses were done using the Kaplan-Meier method, and statistical significance between survival curves was assessed by the log rank test. Disease-specific survival (DSS) was determined from the date of histological-confirmed STS diagnosis.

Multivariate analysis was carried out using the Cox proportional hazards model to assess the specific impact of each pre-treatment variable on survival in the presence of other variables. Only variables of significant value from the univariate analysis were entered into the Cox regression analysis. Probability for stepwise entry and removal was set at 0.05 and 0.10, respectively. The significance level used was p<0.05.

## Results

### Clinicopathological Variables

Demographic, clinical, and histopathological variables are shown in Table 1. Patient age range was 0–91 years (mean 55 years), and 44% of the patients were males. The non-GIST STS comprised 68 undifferentiated pleomorphic sarcoma, 67 leiomyosarcoma, 34 liposarcoma, 20 malignant fibroblastic/myofibroblastic tumors, 16 rhabdomyosarcoma, 16 synovial sarcoma, 13 angiosarcoma, 11 malignant peripheral nerve sheath tumors (MPNST) and 4 other STS. There were 61 low grade STS (24%) and 188 high grade (FNCLCC grade 2 and 3) STS (76%).

**Table 1:** Prognostic clinicopathologic variables as predictors for disease-specific survival soft tissue sarcomas (univariate analysis, log rank test), N = 249.

Characteristic		Patients(n)	Patients(%)	Median survival(months)	5-Year survival(%)	P
**Age**						
≤20 years		20	8	15	40	0.126
21–60 years		113	45	68	52	
>60 years		116	47	30	40	
**Gender**						
Male		110	44	41	46	0.390
Female		139	56	45	45	
**Nationality**						
Norwegian		167	67	63	51	0.011
Russian		82	33	22	34	
**Histology**						
Undifferentiatedpleomorphic sarcoma		68	27	29	40	0.102
Leiomyosarcoma		67	27	45	46	
Liposarcoma		34	14	NR	67	
MF/MFT		20	8	43	50	
Angiosarcoma		13	5	10	31	
Rhabdomyosarcoma		16	6	17	38	
MPNST		11	4	49	45	
Synovial sarcoma		16	6	31	29	
Other STS		4	2	NR	75	
**Tumor localization**						
Extremities		89	36	100	53	0.348
Trunk		47	29	32	44	
Retroperitoneum		37	25	25	38	
Head/Neck		18	7	15	41	
Visceral		58	23	30	42	
**Tumor size**						
≤5 cm		74	30	127	57	0.027
5–10 cm		91	37	44	45	
>10 cm		81	32	28	37	
Missing		3	1			
**Malignancy grade FNCLCC**						
1		61	25	NR	74	<0.001
2		98	39	41	45	
3		90	36	16	26	
**Tumor depth**						
Superficial		17	7	NR	93	<0.001
Deep		232	93	36	42	
**Metastasis at time of diagnosis**						
No	206		83	76	53	<0.001
Yes	43		17	10	10	
**Surgery**						
Yes	228		92	59	50	<0.001
No	21		8	5	0	
**Surgical margins**						
Wide	108		43	NR	62	<0.001
Non-wide	141		57	19	33	
**Chemotherapy**						
No	191		77	52	47	0.424
Yes	58		23	29	40	
**Radiotherapy**						
No	176		71	48	46	0.590
Yes	73		29	38	43	

Abbreviations: MF/MFT, malignant fibroblastic/myofibroblastic tumors; MPNST, malignant peripheral nerve sheath tumor; STS, soft tissue sarcomas; NR, not reached; NOS, non specified.

The treatment option of choice was surgery (n = 228): 118 patients received surgery only; 55 patients received surgery and radiotherapy; 40 patients received surgery and chemotherapy; 13 patients received surgery, radiotherapy and chemotherapy; 2 patients received chemotherapy only; 3 patients received chemotherapy and radiotherapy; 2 patients received radiotherapy only; and16 patients received no therapy. The 5-year survival with non-wide resection margins was 33% and with wide resection margins it was 62%.

### Inter-observer variability

There was good reproducibility between the two investigating pathologists. Their scoring agreement was tested for CD8 and CD20. The IHC scores from each observer were compared using a two-way random effect model with absolute agreement definition. The intra-class correlation coefficients (reliability coefficients, r) obtained from these results were 0.90 for CD8 (P<0.001) and 0.90 for CD20 (P<0.001).

### Univariate analyses

**Figure 2:**
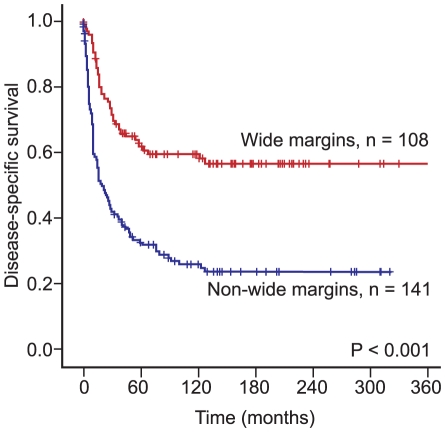
Disease-specific survival curves for patients with wide resection margins compared to patients with non-wide resection margins.

Nationality, tumor size, malignancy grade, tumor depth, metastasis at time of diagnosis, surgery and surgical margins were all significant indicators for disease-specific survival (DSS) in univariate analyses (Table 1, Figure 2). Most of the patients with non-GIST STS who did not survive their disease, died within the first 10 years (120 months). After 10 years almost 60% (n = 108) of the patients with wide resection margins were alive, but only 20% (n = 141) of patients with non-wide resection margins or no surgery (P<0.001), (Figure 2).

**Figure 3:**
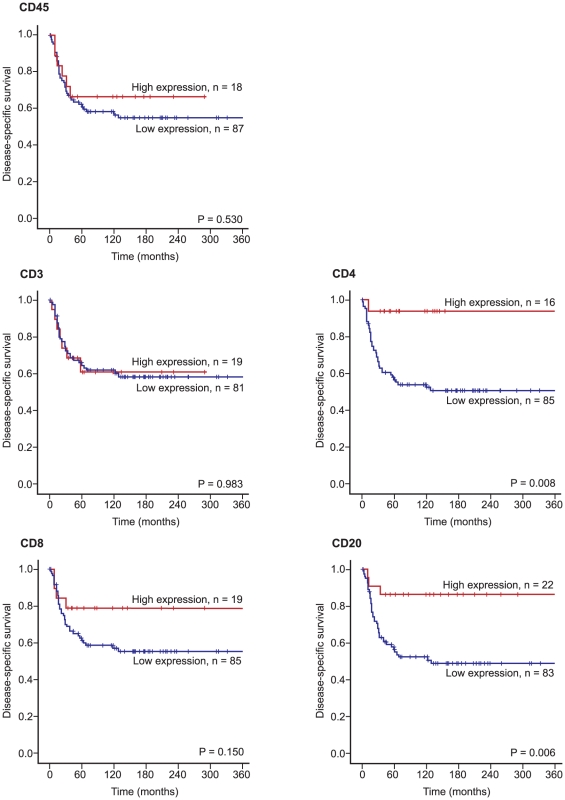
Disease-specific survival curves for CD3+, CD4+, CD8+, CD20+ and CD45+ lymphocytes in STS with wide resection margins.

Furthermore, increasing numbers of CD4+ (P = 0.008) and CD20+ lymphocytes in tumor (P = 0.006) correlated significantly with an improved DSS in patients with wide resection margins (n = 108), (Table 2 and Figure 3). No such relationship was apparent for CD3+, CD8+ and CD45+ lymphocytes. In patients with non-wide resection margins (n = 141) increasing numbers of CD3+ lymphocytes correlated significantly (P = 0.028) with shorter DSS, (Table 2).

**Table 2:** Intratumoral lymphocyte infiltration and their prediction for disease-specific survival in patients with soft tissue sarcomas (univariate analysis; log-rank test), N = 249.

	Non-wide resections margins, n = 141					Wide resection margins, n = 108				
Marker expression	Patients(n)	Patients(%)	Median survival(months)	5-Year survival(%)	P	Patients(n)	Patients(%)	Median survival(months)	5-Year survival(%)	P
**CD 3**										
Low	95	67	26	39	0.028	81	75	NR	64	0.983
High	26	18	15	28		19	18	NR	61	
Missing	20	14				8	7			
**CD 4**										
Low	112	79	23	35	0.474	85	79	NR	57	0.008
High	18	13	12	33		16	15	NR	94	
Missing	11	8				7	6			
**CD 8**										
Low	98	70	22	37	0.349	85	79	NR	61	0.150
High	21	15	26	29		19	18	NR	79	
Missing	22	16				4	4			
**CD 20**										
Low	103	73	26	34	0.447	83	77	NR	55	0.006
High	20	14	11	35		22	20	NR	86	
Missing	18	13				3	3			
**CD 45**										
Low	113	80	21	33	0.745	87	81	NR	61	0.530
High	19	13	25	37		18	17	NR	67	
Missing	9	6				3	3			

Abbreviations: NR, not reached.

Improved survival was seen in patients younger than 60 years (P = 0.005), in tumors of histological grade 1 and 2 (P = 0.011), in tumors less than 5 cm (P = 0.018) and in patients who received chemotherapy (P = 0.024). This was shown through subgroup analysis of patients with high CD20+ lymphocytes in tumor and wide resection margins, Table 3. The same statistical trend was seen for gender, nationality, histology, tumor localization and in patients with or without radiotherapy, but these were not statistically significant (data not shown). There were no significant differences in the expression of the different immunomarkers in the different tumor groups (data not shown).

**Table 3:** Results of subgroup analysis of patients with CD20+ lymphocytes in tumor and wide resection margins, n = 108.

Subgroup	Patients(n)	Patients(%)	Median survival(months)	5-Year survival(%)	P
**Age**					
<60, CD20 Low	49	79	-	56	0.005
<60, CD20 High	11	18	-*	100	
Missing	2	3			
>60, CD20 Low	34	74	NR	54	0.347
>60, CD20 High	11	24	NR	73	
Missing	1	2			
**Histological grade**					
1 or 2, CD20 Low	52	76	-	67	0.011
1 or 2, CD20 High	14	21	-	100	
Missing	2	3			
3, CD20 Low	31	78	28	34	0.155
3, CD20 High	8	20	NR	63	
Missing	1	3			
**Tumor size**					
<5 cm, CD20 Low	28	70	-	63	0.018
<5 cm, CD20 High	10	25	-	100	
Missing	2	5			
>5 cm, CD20 Low	53	78	63	52	0.169
>5 cm, CD20 High	12	18	NR	75	
Missing	3	4			
**Chemotherapy**					
Yes, CD20 Low	24	80	-	82	0.024
Yes, CD20 High	5	17	-	100	
Missing	1	3			
No, CD20 Low	59	76	NR	59	0.080
No, CD20 High	17	22	NR	46	
Missing	2	3			

* Median survival is not computed because all cases are censored.

Abbreviations: NR, not reached.

### Multivariate analyses

Significant demographic, clinicopathological, and lymphocyte infiltrate variables from the univariate analyses were entered into the multivariate Cox regression analysis. An independent positive prognostic factor for improved DSS in patients with wide resection margin was a high number of CD20+ lymphocytes in the tumor (HR 5.5, CI 95% 1.62–18.61, P = 0.006).

Independent negative prognostic variables were Russian nationality (P = 0.020), high malignancy grade (P = 0.016) and metastasis at time of diagnosis (P = 0.001, Table 4). In patients with non-wide resection margins (n = 141) increasing numbers of CD3+ lymphocytes was an independent negative prognostic factor for DSS, (HR 2.2, CI 95% 1.25–3.89, P = 0.007), (Table 4).

**Table 4:** Results of Cox regression analysis summarizing some significant independent prognostic factors in patients with soft tissue sarcomas, N = 249.

	Non-wide resections margins, n = 141			Wide resection margins, n = 108		
Factor	Hazard Ratio	95% CI	P	Hazard Ratio	95% CI	P
**Nationality**						
Norwegian	1.000			1.000		
Russian	1.635	0.978–2.731	0.061	2.246	1.135–4.444	0.020
**Tumor size**			0.428*			0.874*
≤5 cm	1.000			1.000		
5–10 cm	0.826	0.463–1.605		1.217	0.547–2.708	
>10 cm	1.232	0.690–2.199		1.045	0.389–2.806	
**Malignancy grade FNCLCC**			0.024*			0.016*
1	1.000			1.000		
2	1.237	0.611–2.506		3.464	1.081–11.096	0.036
3	2.214	1.108–4.425		5.046	1.656–15.376	0.004
**Metastasis at time of diagnosis**						
No	1.000			1.000		
Yes	3.651	2.081–6.409	<0.001	3.872	1.696–8.836	0.001
**CD3**						
Low	1.000			NIA		
High	2.202	1.245–3.893	0.007			
**CD4**						
Low	NIA			4.126	0.551–30.895	0.168
High				1.000		
**CD20**						
Low	NIA			5.503	1.627–18.606	0.006
High				1.000		

* Overall significance as a prognostic factor.

Abbreviations: NIA, not included in analysis.

## Discussion

In this large scale study, we evaluated whether there is an association between the prevalence of CD3+, CD4+, CD8+, CD20+ and CD45+ lymphocytes in tumors and survival prognosis in 249 non-GIST STS patients. Interestingly, high intensities of CD20+ cells in tumors were an independent positive prognostic factor in patients with wide resection margins.

To our knowledge, this is the first report on CD20 expression in non-GIST STS and the first evidence of its possible clinical relevance in non-GIST STS patients with wide resection margins. This may suggest that CD20+ cells in the tumor are mediating a strong anti-tumor immune response in STS, but this effect is not strong enough to improve survival in patients without wide resection margins.

Activation of the adaptive immune system may suppress malignant cells, whereas activation of various types of innate immune cells may promote tumor growth[14]. The adaptive immunity, orchestrated by antigen-specific T and B-lymphocytes, inhibits tumor growth through both direct killing by cytotoxic T-lymphocytes, and a combination of cytokine and antibody mediated tumor cell lysis[14]. Cancer infiltration by tumor reactive T-lymphocytes is required for efficient tumor eradication[15]. However, cancer cells can escape the immune system in several ways including suppression of cytotoxic T-cells, by regulatory T-cells and by accumulation of myeloid suppressor cells[15]–[17].

Tumor-infiltration CD3+ cells are reported to be strongly associated with favorable prognosis in epithelial tumors in several studies[18]–[22]. The CD3+ cell is an independent positive predictor of response to neoadjuvant chemotherapy in breast cancer[23]. Low numbers of CD3+ lymphocytes predicted shorter disease-free survival in colon cancer[24] and cervical cancers[25]. However, T-cell parameters including CD3 values showed no correlation with survival in cases of metastatic ovarian carcinoma[26]. Accordingly, we did not find any such association in our mesenchymal material in patients with wide resection margins, but CD3 was a negative prognostic factor in patients with non-wide resection margins.

The role of CD4+ T and B lymphocytes is controversial in many cancers including STS; CD4+ cells in the absence of the CD8+ cytotoxic T cells are critical and sufficient for NKT cell-dependent rejection of experimental tumours[27]. In lung cancer the prognostic impact of CD4 is controversial[28], [29], but in our material CD4+ cells were a positive prognostic factor in univariate analyses.

CD8+ cells in malignant tumors have been associated with a better survival in many types of cancer including: non-small cell lung carcinoma; carcinomas of the endometrium, bile duct, colon, oesophagus, urothelium; and uveal melanoma and follicular lymphoma[28], [30]–[37]. However, the role of CD8+ lymphocytes in STS is controversial and most of the studies contain relatively few cases. There was a positive correlation between a high density of CD4+ and CD8+ lymphocytes in stroma and improved disease-specific survival in non-small cell lung cancer[28]. In our material CD8 was not a statistically significant factor (P = 0.15).

CD20+ cells are associated with a better survival in lung cancer, cervical cancer, prostate cancer and ovarian cancer[25], [28], [38]–[40]. CD20+ B-cells in metastatic lymph nodes are associated with favourable outcome in patients with oro- and hypopharyngeal carcinoma[41]. On the other hand, B-cell infiltration detected by flowcytometry with CD19 were correlated with unfavourable outcome in metastatic ovarian carcinoma[26]. In our material high density of CD20+ lymphocytes was an independent positive prognostic indicator.

In cervical cancer no significant impact of CD45+ cells were seen [25], neither was it in non-GIST STS in this study.

The optimal chance for curing localized STS is based on wide resection surgery. Given that of the majority of STS patients succumb to this disease within 5 years, there is an apparent need for better systemic therapy including novel molecularly targeted therapies[42]. In our study, there was a 33% 5-year survival in the group with non-wide resection margins and 62% of those with wide resection margins.

Among STS patients who have had wide resection margins, it will be essential to identify those who will relapse and succumb this disease as these patients may benefit from adjuvant therapy, including immunotherapy. Until now adjuvant chemotherapy has been controversial due to inadequate selection criteria.

The human immune system contains specialized cells that are able to eliminate cancer cells[26], and tumor-infiltrating B-cells are able to produce tumor-specific antibodies[43]. Through external stimulation of the immune response, these cells may have the potential to aid the immune system in destroying single tumor cells and micro-metastases after surgery. This topic is investigated in the ongoing international osteosarcoma protocol EURAMOS where those who respond well to chemotherapy are randomized to receive interferon or no interferon, in an attempt to improve the immune response (http://www.ctu.mrc.ac.uk/euramos/).

In conclusion, high density of CD20+ lymphocytes in STS with wide resection margins is an independent positive prognostic indicator for these patients. Further research to define if CD20+ cells can modify tumors in a way that reduces disease progression and metastatic potential is needed.
